# Recalcitrant intussusception: exploring potential associations with *Helicobacter pylori* infection - a case report and literature review

**DOI:** 10.1186/s13099-024-00621-z

**Published:** 2024-06-01

**Authors:** Kuan-Chieh Wang, Chun-Hao Chu, Che-Ming Chiang, Fu-Ruei Zeng, Ching-Wen Huang, Chien-Ming Lin

**Affiliations:** 1 School of Medicine, National Defense Medical Center, Taipei, Taiwan; 2 Department of General Medicine, Tri-Service General Hospital, National Defense Medical Center, Taipei, Taiwan; 3 Department of Pediatrics, Zuoying Armed Forces General Hospital, Kaohsiung, Taiwan; 4 Department of Pediatrics, Tri-Service General Hospital, National Defense Medical Center, No. 325, Sec.2, Chenggong Rd., Neihu District, Taipei, 114 Taiwan

**Keywords:** Intussusception, Helicobacter pylori, Henoch-schönlein purpura, Triple therapy

## Abstract

**Background:**

Intussusception, a common cause of abdominal pain in children, often lacks clear underlying causes and is mostly idiopathic. Recurrence, though rare, raises clinical concerns, with rates escalating after each episode. Factors like pathological lead points and Henoch-Schönlein purpura (HSP) are associated with recurrent cases. On the other hand, the prevalence of Helicobacter pylori (*H. pylori*), often asymptomatic, in children has been declining. Although its infection is reported to be linked with HSP, its role in recurrent intussusception remains unexplored. Further research is needed to understand the interplay among *H. pylori* (culprit pathogen), HSP (trigger), and intractable intussusception so as to develop effective management strategies.

**Case presentation:**

A two-year-old girl experienced four atypical episodes of intussusception at distinct locations, which later coincided with HSP. Despite treatment with steroids, recurrent intussusception persisted, suggesting that HSP itself was not a major cause for intractable presentations. Subsequent identification of *H. pylori* infection and treatment with triple therapy resulted in complete resolution of her recalcitrant intussusception.

**Conclusion:**

This instructive case underscored a sequence wherein *H. pylori* infection triggered HSP, subsequently resulting in recurrent intussusception. While *H. pylori* infection is not common in young children, the coexistence of intractable intussusception and steroid-resistant recurrent HSP necessitates consideration of *H. pylori* infection as a potential underlying pathogen.

## Background

Intussusception is the second most common cause of abdominal pain in children but the underlying disease contributing to it is less addressed. Typically, a significant proportion of intussusception cases (75–90%) are classified as idiopathic, while a smaller percentage (approximately 2–8%) can be attributed to lead points. Recurrent intussusception, though relatively uncommon at 8–15%, poses a significant concern in clinical practice. The recurrence rate, escalating with each subsequent attack post-reduction, is reported at approximately 15.7% after the first episode and can reach around 70% after the third [1]. Several factors are associated with recurrent intussusception, including age above two years, symptom duration exceeding 48 h, presence of pathological lead points, and Henoch-Schönlein purpura (HSP) [2]. Therefore, if the underlying causes cannot be identified, recurrent intussusception may persist, resulting in intractable abdominal pain.

A potential association between Helicobacter pylori *(H. pylori)* infection and HSP occurrence has been reported [3], though the prevalence of *H. pylori* infection in children, approximately 32.3%, has been gradually decreasing. Household transmission, including oral-to-oral, fecal-oral, and gastrointestinal routes, is the primary mode. Approximately 90% of *H. pylori* infection cases are asymptomatic [4], but its association with recurrent intussusception remains unexplored to date. Furthermore, there have been no reported cases of untreated *H. pylori* infection deteriorating to the extent of causing HSP, which subsequently leads to intractable intussusception.

We present a two-year-old girl with four unusual episodes of intussusception occurring at different locations. Concurrently, she exhibited the coexistence of HSP, and despite steroid therapy, recurrent intussusception persisted, indicating that latent etiologies associated with HSP might contribute to the recurrence. Subsequently, *H. pylori* infection was detected, and her recalcitrant intussusception completely resolved after triple therapy. To the best of our knowledge, this is the first reported case of its kind, highlighting the significance of recognizing the latent *H. pylori* infection causing HSP in children with intractable intussusception.

## Case presentation

**Fig. 1:**
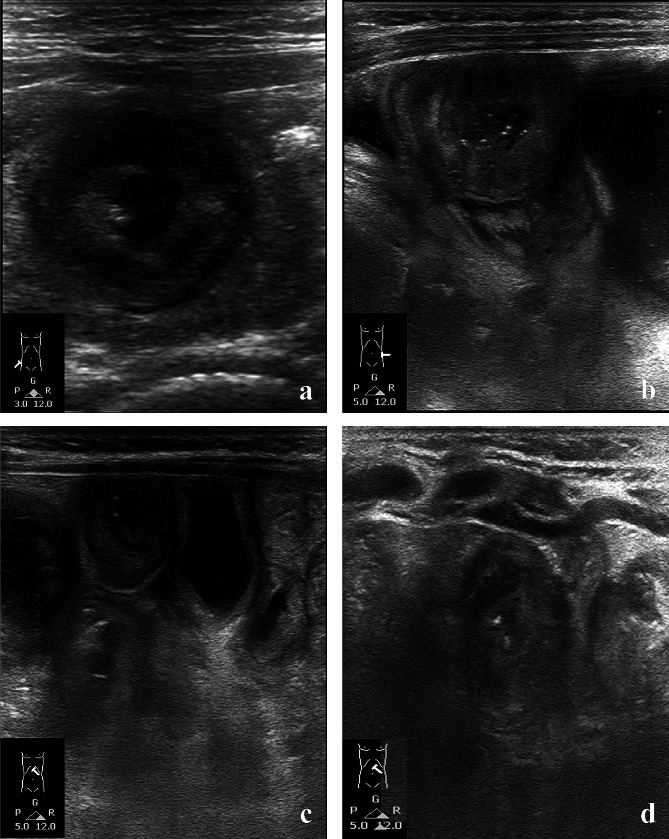
Diverse recurrent loci of recalcitrant intussusception on sonography. **(a)** Initial image of right lower quadrant **(b)** Subsequent image of left middle quadrant **(c)** Third image of left upper quadrant **(d)** Fourth image of left upper quadrant

**Fig. 2:**
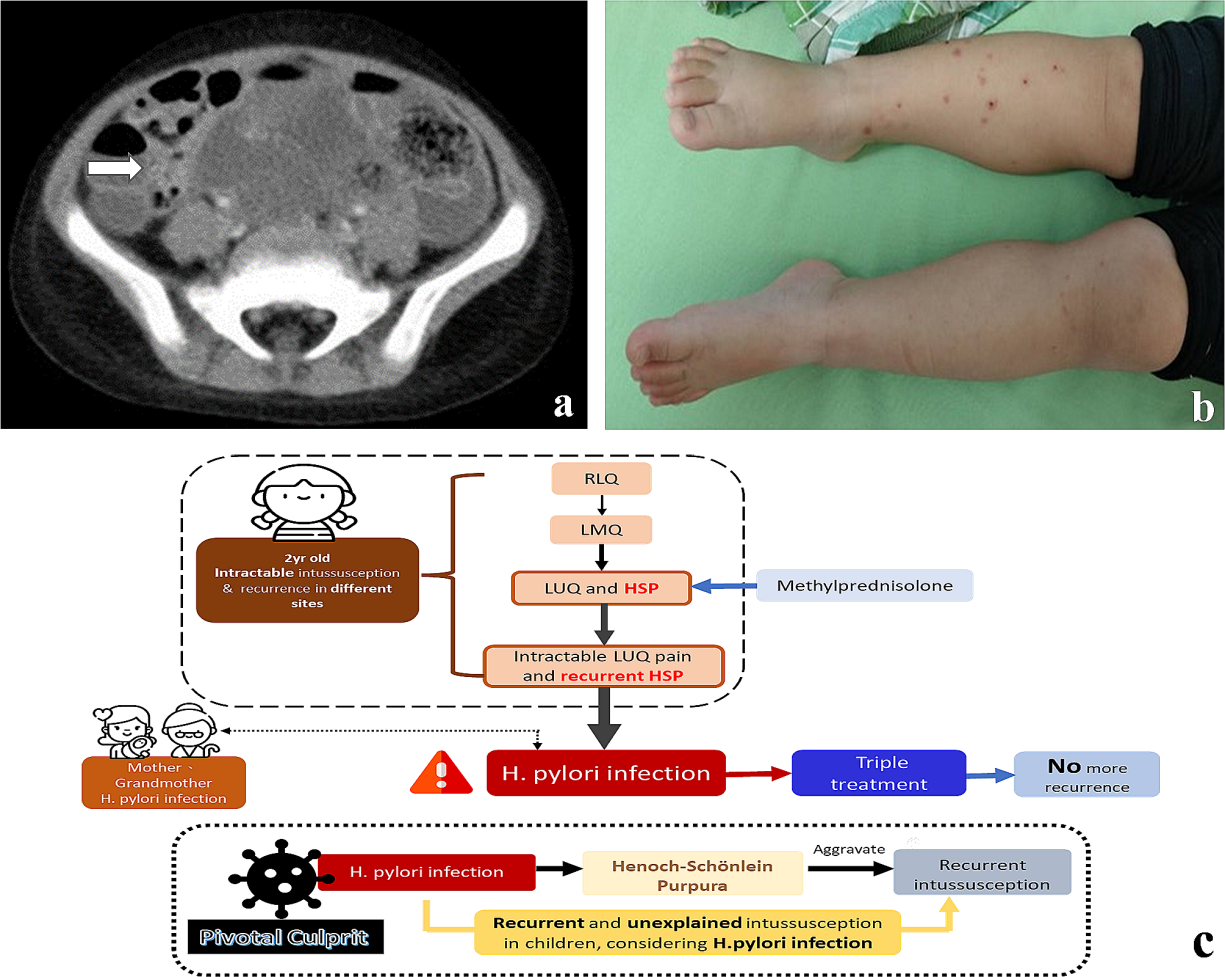
Exploring clinical manifestations and images of the index patient with recurrent intussusception. **a**. CT image showed enlarged mesenteric lymph nodes. **b**. Multiple purpuric macules and papules over lower limbs. **c.** Clinical role of H. pylori infection as a culprit in recalcitrant intussusception and HSP

A two-year-old girl has no history of easy lethargy, hypotonia, electrolyte imbalance, or hyperpigmented skin lesions. She presented to the emergency department with intermittent, crampy, progressive abdominal pain, accompanied by inconsolable crying. Milk, wheat, shellfish, peanuts, and fish were not consumed in the past month. Abdominal ultrasonography revealed a mass lesion with a target sign in the right lower abdomen **(**Fig. 1a**)**, which was immediately remitted by hydrostatic reduction. Despite successful reduction, the reappearance of abdominal pain on the next day confirmed recurrent intussusception in the left abdomen **(**Fig. 1b**)**. Computed tomography scan was performed to exclude structural anomaly, revealing only bowel wall thickening and enlarged mesenteric lymph nodes **(**Fig. 2a**)**. Despite successful reduction again, a third episode of intussusception over the left upper quadrant (LUQ) was found one week later **(**Fig. 1c**)**. Concurrently, she presented with multiple palpable purpuras over lower limbs and arthralgia at the same time **(**Fig. 2b**)**, compatible with clinical diagnosis of HSP. Laboratory data showed no proteinuria, thrombocytopenia, or coagulopathy. Consequently, a 10-day course of intravenous methylprednisolone was administered due to HSP. Two weeks after discharge, the fourth episode of intussusception over the LUQ, accompanied by a flare-up of purpura on the lower limbs, was observed **(**Fig. 1d**)**. Intravenous steroids were re-administered. A small bowel series revealed no occult lead points. Given the unusual recurrence with diverse localization of intussusception, an infectious survey was conducted. No common respiratory virus infection was detected on the FilmArray Respiratory Panel. However, the stool antigen test eventually demonstrated *H. pylori* infection. As *H. pylori* infection might be associated with intussusception per se or contribute to HSP-aggravating intussusception, triple therapy comprising a proton pump inhibitor, amoxicillin and metronidazole was administered for 14 days. The negative *H. pylori* status was confirmed by stool antigen detection 2 months after therapy cessation. The complete resolution of HSP was also noted in the wake of *H. pylori* eradication. Notably, the patient’s mother and grandmother were also infected with *H. pylori*. Currently, at 4 years old, there has been no recurrence of intussusception.

## Discussion and conclusions

### Association between *H. Pylori* and recurrent intussusception

Intussusception is a commonly encountered condition in children, with approximately 90% of cases classified as idiopathic [5]. Indeed, factors such as food allergies [6], virus infections [7], and genetic predispositions [8–10] can all contribute to recurrent intussusception. Moreover, recurrence of intussusception occurs in approximately 10% of children following successful non-surgical reduction, with a higher risk observed, especially in children aged above one year [11]. In our case, detailed history taking revealed no prior allergic history or recent consumption of any potentially relevant foods within the past month. Testing with the FilmArray Respiratory Panel did not identify common culprits such as adenovirus or other respiratory viruses typically linked to recurrent intussusception. Although genetic diseases such as *SAMD9* mutation, Peutz-Jeghers syndrome, and Schaff-Yang syndrome may be associated with intussusception [8–10], our case did not exhibit typical presentations of these diseases. Notably, the case patient presented with the unusual occurrence of four episodes of intussusception, each manifesting at different recurrent locations, suggesting the presence of underlying unresolved causes. Although *H. pylori* is not commonly associated with intussusception and has a higher prevalence in children aged 7–9 years [12], the present young case, experiencing intractable intussusception resolved by eradicating *H. pylori* within the family, highlights its potential pathogenic role in atypical and recalcitrant cases.

### Association between *H. Pylori* and recurrent HSP

On the other hand, *H. pylori* is the second most common pathogen associated with HSP, a systemic immunoglobulin A (IgA) vasculitis characterized by abdominal pain [13]. Among individuals infected with *H. pylori*, the presence of elevated IgA and cryoglobulin can be observed, eventually leading to an increased formation of immune complexes and overactivated IgA [14, 15]. In addition, *H. pylori* infection triggers the secretion of pro-inflammatory cytokines, including interleukin (IL)-6, IL-12, and tumor necrosis factor-alpha (TNF-α) [16]. The formation of immune complexes and subsequent autoimmune responses are thought to be crucial in *H. pylori*-associated HSP [13]. In a nutshell, *H. pylori* infection significantly increases the risk of developing HSP, which, in turn, aggravates recurrent intussusception. Therefore, it has been reported that clinical symptoms of HSP can be alleviated with eradication therapy for *H. pylori* [17], as seen in the present case exhibiting well-resolved symptoms of intractable intussusception and HSP after triple therapy.

### Both recurrent HSP and latent *H. pylori* contribute to unsolved intussusception

Histologically, HSP is characterized by atypical glycosylation of IgA1, notably the presence of GalNAc as the terminal glycosylation, resulting in immune complexes deposition [18, 19]. HSP can impact multiple organs, including the skin, kidneys, and gastrointestinal system, with noteworthy involvement often leading to intussusception, particularly in children around 6 years old [20]. On the other hand, the presence of pathological lead points is a significant predictor for recurrent intussusception [21]. In the present case, the two-year-old girl experienced recurrent and intractable intussusception initially thought to be related to HSP alone but showed a poor response to steroid treatment. These unique findings and clinical course all suggest the possibility of latent etiologies behind HSP causing intussusception. While HSP can be considered a significant lead point, given its propensity for inflammation of blood vessels and causing bleeding within the submucosal and subserosal layers [22, 23], underlying HSP etiologies should be emphasized in clinical practice when recurrent intussusception coexists with HSP.

### Our unique manifestations compared with reported cases

Table 1 shows a comprehensive comparison of five cases related to *H. pylori*-associated intussusception. Notably, the majority of documented cases involved adults rather than children. None of these cases resembled our recurrent intussusception, with each episode occurring in different locations. While Sana Abourazzak et al. reported a four-year-old girl with *H. pylori*-associated intussusception, the presence of HSP manifestations was unique in our case. The distinctive manifestations suggest that the coexistence of *H. pylori*-associated HSP and intractable intussusception is rare. Furthermore, the recurrence of HSP with a poor response to intravenous steroids strongly indicates the presence of underlying culprits, potentially leading to recurrent intussusception. Ultimately, the eradication of *H. pylori* and the subsequent resolution of HSP underscore the clinical importance of identifying *H. pylori* infection as a potential cause for unexplained and recalcitrant intussusception.

While our case lacked potential causes like occult lead points, viral infections and food allergies, establishing a direct link between *H. pylori* and recurrent intussusception from a single case report is challenging. However, the pattern of recurrent intussusception coinciding with *H. pylori* infection, which resolved in the wake of eradication, and the absence of HSP recurrences post-eradication, supporting the biological plausibility and coherence of our observations. Yet, further large-scale prospective studies and comprehensive genetic investigations are still needed to confirm whether this association is causal or coincidental.

**Table 1:** Comparison of clinical features between published *H. pylori*-associated intussusception patients and current case

No.	Study	Sex	Age (y/o)	Symptom to Dx (day)	Location	H. pylori	HSP	Clinical presentation	Recurrent time	Tx
1	Current case	F	4	24 days	RLQ to LMQ to LUQ	+	+	Intermittent, crampy, progressive abdominal pain, accompanied by inconsolable crying	4	Methylprednisolone (ineffective)/ Triple therapy
2	Sana Abourazzak et al.,2022 [24]	F	4	N/A	UQ	+	-	Pallor, striking epigastric pain, nausea and vomiting	0	Operation
3	Shilpa Lingala et al., 2018 [25]	M	38	2 days	RUQ and RLQ	+	-	Intermittent abdominal pain, nausea, and vomiting	0	Triple therapy
4	TH Hung et al., 2022 [26]	M	64	N/A	UQ to RUQ	+	-	Intermittent epigastric discomfort, dyspepsia, decreasing appetite and melena	0	Whipple procedure with *H. pylori* eradication therapy
5	Wan Najmi, W D et al.,2016 [27]	M	42	~ 3 months	N/A	+	-	Intermittent recurrent vomiting and weight loss	0	Partial gastrectomy with *H. pylori* eradication therapy

**Abbreviation**: Dx, diagnosis; *H. pylori*, Helicobacter pylori; HSP, Henoch-Schönlein purpura; LMQ, left middle quadrant; LUQ, left upper quadrant; N/A, not applicable; RLQ, right lower quadrant; RUQ, right upper quadrant; Tx, treatment; UQ, upper quadrant; y/o, year old

## Conclusion

The present case highlighted a sequence of events wherein *H. pylori* infection led to HSP, subsequently causing recurrent intussusception at various locations. After successful *H. pylori* eradication therapy, the patient’s condition improved significantly. Therefore, given the coexistence of intractable intussusception with diverse locations and recurrent HSP resistant to typical steroid treatment, it is crucial to consider the potential role of *H. pylori* infection as an underlying culprit **(**Fig. 2c**)**.

## Data Availability

No datasets were generated or analysed during the current study.

## Abbreviations

CT: Computed tomography
Dx: Diagnosis
GalNAc: N-Acetylgalactosamine
H. pylori: Helicobacter pylori
HSP: Henoch-Schönlein purpura
IgA: Immunoglobulin A
LMQ: Left middle quadrant
LUQ: Left upper quadrant
N/A: Not applicable
RLQ: Right lower quadrant
RUQ: Right upper quadrant
Tx: Treatment
UQ: Upper quadrant
y/o: Year old
